# Host Plant Preferences and Survival of the Native Australian Spittlebug, *Bathyllus albicinctus* Erichson (Hemiptera: Cercopoidea)

**DOI:** 10.3390/biology15110886

**Published:** 2026-06-03

**Authors:** Duncan Jaroslow, Narelle Nancarrow, Mark Blacket, Cait Selleck, Kudzaishe Precious Mavende, Piotr Trębicki

**Affiliations:** 1 AgriBio, Agriculture Victoria, Bundoora, VIC 3083, Australia; 2 Grains Innovation Centre, Agriculture Victoria, Horsham, VIC 3400, Australia; 3 Applied BioSciences, Macquarie University, Sydney, NSW 2109, Australia

**Keywords:** xylem-feeding insects, nymph development, no-choice host experiment, host suitability

## Abstract

The xylem feeder *Bathyllus albicinctus* (Aphrophoridae: Cercopoidea: Hemiptera) has been identified as one of the most abundant and widely distributed cercopoid insects in Australia. This native Australian species might potentially vector *Xylella fastidiosa*, an exotic and deadly plant pathogen, if it were to arrive in Australia. Previous surveys indicated that *B. albicinctus* is associated with a wide range of plants, further elevating its impact as a potential disease vector. However, it is unknown if all the associated plants are equally viable hosts. Due to the variety of plant records and distribution across the southern half of Australia, it was expected that *B. albicinctus* would successfully feed and survive on a taxonomically diverse range of plants. In the present study, a small-scale no-choice feeding-controlled glasshouse experiment was completed where insect nymphs were supplied with plants that are economically significant and susceptible to *X. fastidiosa*. In this experiment, we monitored the maturation and longevity of *B. albicinctus*, specifically by comparing insect cohorts distinguished by nymph size, host species, or feeding season. This study documented important interactions of life-history factors for *B. albicinctus*, including suitability of host plant species among insect sex and season. This could indicate that the ability for *B. albicinctus* to spread *X. fastidiosa* may vary with host plant species and time of year. These findings provide a vital knowledge framework for future research and surveys to consider when implementing control efforts or tracking plant pathogens that may be spread by *B. albicinctus*.

## Simple Summary

Worldwide xylem-feeding insects in the Cercopoidea group are responsible for spreading the deadly plant pathogen *Xylella fastidiosa*, resulting in significant damage to economically significant crop plants. Although this exotic pathogen has not arrived in Australia, potential arrival and establishment in economically significant crops might be facilitated by the abundant and widely distributed local Cercopoidea insects, namely *Bathyllus albicinctus*. Our research reveals variation in the suitability of select plant species as hosts for *B. albicinctus* in a no-choice glasshouse experiment. Insect longevity and growth varied with host plant species, insect sex, and season. This study provides insights into the host preferences and survival of a common Australian insect in relation to its potential to spread the exotic pathogen *Xylella fastidiosa* prior to any incursion.

## 1. Introduction

*Xylella fastidiosa* (Xanthomonadaceae) is a significant threat to both agricultural and environmental systems and has been recorded infecting 727 species of plants across 91 families [1], many of which are economically significant (e.g., citrus, grape, olive, coffee, pine, and sorghum). Incredibly damaging *X. fastidiosa* outbreaks have occurred in these crops across several continents, including regions in Europe [2], the Americas [3,4], Asia [5], and the Middle East [6], generating urgency to better understand how this pathogen is spread and which plant species are most vulnerable. *X. fastidiosa* bacteria inhabit the water transport vessels (xylem) of vascular plants [7] and produce waste biofilm that can halt water movement within xylem vessels [8,9], which ultimately leads to plant death [10]. *Xylella fastidiosa* is spread between plants by xylem-feeding Auchenorrhyncha within Hemiptera, notably polyphagous Cercopoidea (spittlebugs) and a small group of xylem-feeding Cicadellidae (leafhoppers). Transmission of *X. fastidiosa* into plant tissue occurs from insect stylets into xylem vessels during insect feeding [11,12] and specifically during ‘egestion’ (insect backwash when tapping the xylem) [13]. This mechanical transmission enables *X. fastidiosa* to be distributed by a wide range of mobile xylem-feeding insects, thus exposing a wide range of plants to these deadly bacteria. Understanding the community of potential *Xylella* vectors in Australia is of great benefit to the pre-emptive and preparative management efforts for the future possibility of *X. fastidiosa* arriving in Australia.

The primary vectors of *X. fastidiosa* vary across continents and are often abundant and common species. In California the primary vectors include sharpshooters such as *Graphocephala atropunctata* Signoret (Hemiptera: Cicadellidae) and *Homalodisca vitripennis* Germar (Hemiptera: Cicadellidae) [3], whereas, in Morocco, *X. fastidiosa* spread is expected to be most associated with *Philaenus tesselatus* Horvárth (Hemiptera: Aphrophoridae) [14]. In Europe, the primary vectors include *Philaenus spumarius* L. (Hemiptera: Aphrophoridae) and *Neophilaenus campestris* Fallén (Hemiptera: Aphrophoridae) [15], with *P. spumarius* regarded as the key vector [16]. The capacity of *P. spumarius* to vector *X. fastidiosa* is relatively high due to its abundance, broad distribution, and polyphagous nature [17]. With an increased focus on managing *X. fastidiosa* outbreaks through vector control, there is mounting evidence that vegetation type and climate conditions can be incredibly important for predicting fluctuations in the abundance of vectors and their disease transmission risk (e.g., *Philaenus spumarius*) [18,19]. Although *X. fastidiosa* is not currently in Australia, the poor understanding of the Australian cercopoid community limits the preparedness for, and capacity to respond to, a *X. fastidiosa* incursion. This preparedness and response capacity may be greatly improved by investigating the feeding habits and seasonal abundance of polyphagous Cercopoidea in Australia.

**Figure 1:**
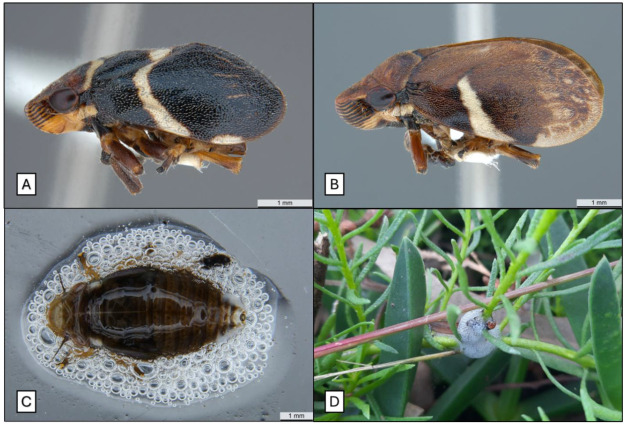
*Bathyllus albicinctus*, lateral view of (**A**) adult male and (**B**) adult female, (**C**) dorsal view of nymph (immature insect) with spittle, and (**D**) early instar nymph entering an existing spittle mass on *Myoporum parvifolium*.

*Bathyllus albicinctus* Erichson (Hemiptera: Aphrophoridae) (Figure 1) is among the most common species of Cercopoidea in Australia and has established in New Zealand [20,21]. Transmission and establishment of *X. fastidiosa* require a capable herbivore vector and viable host plant for that herbivore [13]. Outside of Australia some host plants of *B. albicinctus* (e.g., *Westringia fruticosa*, *Acacia* spp., and *Olea europaea*) are documented as susceptible to *X. fastidiosa* [1,22]. Currently, *B. albicinctus* is predicted to be likely capable of acting as a vector of *X. fastidiosa* due to its abundance, distribution, polyphagous diet, and taxonomic relatedness to *P. spumarius*. An improved understanding of *B. albicinctus*, and Australian Cercopoidea in general, would greatly improve management efforts for effective surveillance and exclusion of *X. fastidiosa* from Australia. Documenting the suitability of putative host plants of *B. albicinctus* can provide an indication of which plants this insect would be most likely to infect with *Xylella*. Investigating *B. albicinctus* insect development, host preference, and longevity provides vital background knowledge to effectively monitor for *X. fastidiosa* and prevent its establishment and potential spread in Australia.

To better understand the development, feeding activity, and longevity of *B. albicinctus,* we conducted a controlled glasshouse experiment, monitoring *B. albicinctus* contained on one of four plant species maintained in pots. Due to the role of seasonality in insect activity and *Xylella* transmissibility [4], *B. albicinctus* nymphs were monitored in two discrete season-based cohorts: spring and summer. The host plants used in this study are known to be susceptible to *Xylella fastidiosa* [1] and are also either widespread across, or are economically significant to, Australia. It was expected that this study would highlight the season and plant species that present the greatest *Xylella* transmission risk by documenting the associated variations in insect activity.

## 2. Materials and Methods

### 2.1. Maturation and Longevity of Bathyllus albicinctus on Host Plants Susceptible to Xylella fastidiosa

#### 2.1.1. Growth Conditions

Grapevine (*Vitis vinifera*: Vitaceae), olive (*Olea europaea*: Oleaceae), orange (*Citrus × sinensis*: Rutaceae), and common sow thistle *(Sonchus oleraceus*: Asteraceae) plants were grown in a laboratory, which was kept at 22 °C, and had access to a natural light cycle. Sow thistle plants found with *B. albicinctus* around Horsham, Victoria, Australia, were initially carefully translocated from the field to laboratory conditions and were intended for use as a ‘positive control host plant’ group. However, the sow thistle plants did not adjust to lab conditions well, likely due to translocation stress.

#### 2.1.2. Insect Collection and Host Plants

The 2nd to 4th instar *B. albicinctus* nymphs were field-collected from self-propagated plant vegetation in and around Horsham, Victoria. To assess development and longevity, *B. albicinctus* nymphs were collected from coastal rosemary (*Westringia fruticosa*), sow thistle (*Sonchus oleraceus*), and an unidentified Lamiaceae plant. Insects were individually placed on the petiole/leaf of an orange tree (*Citrus* × *sinensis*), two varieties of olive (*Olea europaea*), four varieties of grapevine (*Vitis vinifera*), and field-transplanted sow thistle (*Sonchus oleraceus*) (Table 1). A clip cage [23] was then placed over the top of individual nymphs. Two trials were conducted for this experiment at The Grains Innovation Park, Horsham, Victoria, beginning with transferring *B. albicinctus* nymphs to their respective host plants. Trial 1 began in October 2022, and Trial 2 began in December 2022, when *B. albicinctus* nymphs could be collected for use in these experiments.

#### 2.1.3. Insect Activity

**Figure 2:**
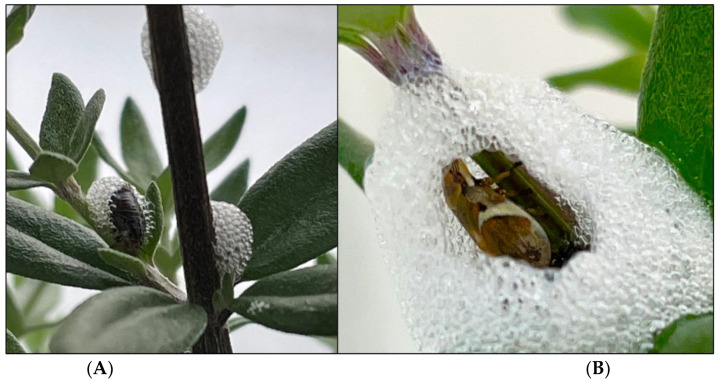
(**A**) *Bathyllus albicinctus* nymphs feeding on *Westringia fruticosa* and excreting watery spittle, seen as bubbles. (**B**) *Bathyllus albicinctus* adult female resting under spittle ‘igloo’.

Instar development, presence of spittle excretions (Figure 2), adult emergence, and insect mortality were recorded as metrics for comparing the development and longevity of *B. albicinctus*. The weight of insects, and sex for those that reached adulthood, were also recorded as indicators of development or sex-based divergence.

### 2.2. Statistical Analyses

Statistical analyses were performed using RStudio (version 4.1.0), with appropriate data manipulations made using the ‘dplyr’ package [24]. Histogram visual representations were prepared in Microsoft Excel (version 2403), and visual representations of regression models were prepared within R, in part using the R package ‘ggplot2’ [25].

The maximum and mean number of days before *B. albicinctus* in clip-cages matured into adults, or perished, were compared descriptively and then discretely among host plant species and between Trial 1 and Trial 2. Kruskal–Wallis independent samples test of significant difference was used to detect differences (α = 0.05) in *B. albicinctus* activity within trial cohorts based on host plant species, and a Dunn test was used for post hoc analysis [26] to indicate which group pairs were statistically different (null hypothesis rejected if *p* ≤ α/2). Welch’s two-sample test was used to detect differences (α = 0.05) between Trial 1 and Trial 2. This test was also used to detect differences in insect weight between male and female insects and between trials. Kruskal–Wallis test was used to detect differences (α = 0.05) in insect weight among male, female, and nymphal *B. albicinctus*, and Dunn test was used to detect differences across life stages (null hypothesis rejected if *p* ≤ α/2).

Regression analysis was also used to identify statistically significant correlations among insect longevity, number of days spent excreting spittle, and the number of days before reaching adulthood. Regression analyses were inclusive of insects across month or host groupings.

## 3. Results

### 3.1. Bathyllus Maturation in Spring and Summer

**Figure 3:**
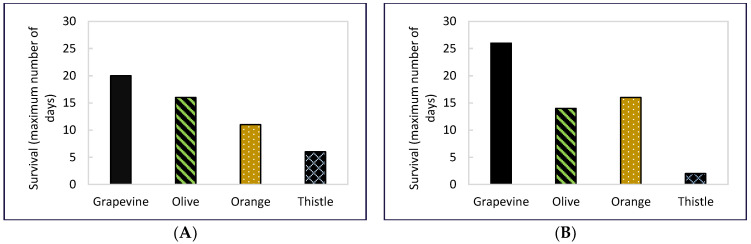
The maximum number of days that *Bathyllus albicinctus* survived on each host type in (**A**) Trial 1 and (**B**) Trial 2.

Initially, the mortality rates that were recorded in the first few days of Trial 1 (starting in October 2022) and Trial 2 (starting in December 2022) were high (personal observation), but maximal survival duration reached 20 days in Trial 1 and 25 days in Trial 2 (both on grapevine) (Figure 3). The maximal survival of *B. albicinctus* ranged from 11 to 16 days for olive and orange, respectively, across both experiments. The maximal survival was six days on sow thistle, which was the lowest of the four hosts, although many of these insects were also initially collected from sow thistle.

**Figure 4:**
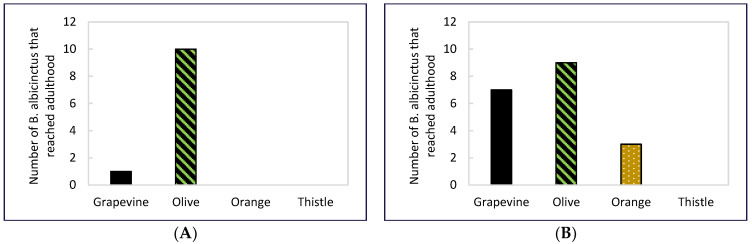
The number of *Bathyllus albicinctus* that reached adulthood on each host type in (**A**) Trial 1 and (**B**) Trial 2.

*B. albicinctus* in Trial 1 only reached adulthood on olives and grapevine. Adults in Trial 2 were recorded for grapevine, olive, and orange. In both trials, olive had the most nymphs that reached adulthood. *B. albicinctus* did not reach adulthood on oranges in Trial 1 or on sow thistle in either trial (Figure 4).

### 3.2. Maturation of Bathyllus on Host Plants Susceptible to Xylella

Across the October and December trials, statistical differences were not found between the host plant types (Table A1) for *B. albicinctus* longevity (Kruskal–Wallis: χ^2^ = 6.354, *n* = 122, *p* = 0.1) or time to adulthood (Kruskal–Wallis: χ^2^ = 1.829, *n* = 27, *p* = 0.61). Across the trials, differences based on host plant species were detected in the number of days *B. albicinctus* produced spittle excretions (Kruskal–Wallis: χ^2^ = 9.748, *n* = 122, *p* = 0.02). The post hoc analysis (Table A2) revealed that insects feeding on olives would produce spittle more frequently than insects feeding on citrus-oranges (Dunn test: *p* = 0.007) and grapevine (Dunn test: *p* = 0.003). 

**Figure 5:**
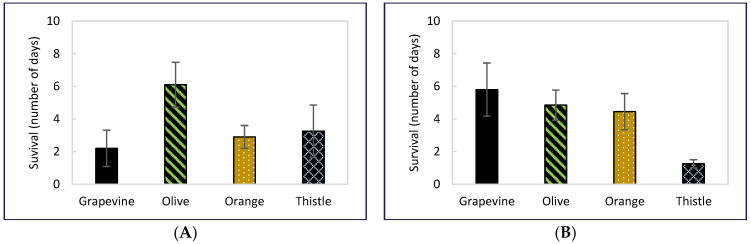
The mean number of days that *Bathyllus albicinctus* survived on each host type in (**A**) Trial 1 (grape *n* = 18; olive *n* = 20; orange *n* = 20; thistle *n* = 4) and (**B**) Trial 2 (grape *n* = 20; olive *n* = 20; orange *n* = 20; thistle *n* = 4).

In Trial 1, there were no statistically significant differences in insect survival when testing across the host plant species (Kruskal–Wallis: χ^2^ = 7.4401, *n* = 60, *p* = 0.06; Figure 5A). The independent samples test did not indicate differences in insect survival between the plants in Trial 2 (Kruskal–Wallis: χ^2^ = 3.7115, *n* = 60, *p* = 0.29). Due to the non-significant results of these Kruskal–Wallis tests, caution is needed when interpreting the variation visualised in Figure 5. For Trial 2, *B. albicinctus* on sow thistle survived for approximately half the number of days compared to insects on olive (Figure 5B). Differences in insect survival were not found between grapevine, orange, or olives within Trial 2 (Table A3). Contrastingly, *B. albicinctus* feeding on grapevine had the shortest survival time in Trial 1 and the longest in Trial 2.

Differences in the number of days insects spent producing spittle may be present based on host plant type in Trial 1 (Kruskal–Wallis: χ^2^ = 7.836, *n* = 58, *p* = 0.05) but not in Trial 2 (Kruskal–Wallis: χ^2^ = 3.712, *n* = 60, *p* = 0.29). The pairwise comparison did, however, reveal some differences for insects in the same cohort (Table A4) reared on different host plants. In Trial 1, *B. albicinctus* feeding on olives produced spittle more often than *B. albicinctus* feeding on grapevines (Dunn test: *p* = 0.006). Differences in the number of days it took to reach adulthood were not detected between the host plant groups within the October (Kruskal–Wallis: χ^2^ = 0.603, *n* = 16, *p* = 0.74) or December (Kruskal–Wallis: χ^2^ = 1.5501, *n* = 16, *p* = 0.46) trial groups. The statistical comparison of the days survived, days involving spittle excretions, and the number of days it took to reach adulthood did not reveal differences between Trial 1 and Trial 2 or between host plant species (Table A3).

Discrete tests between the weight and sex of *B. albicinctus* and the weight of *B. albicinctus* and starting experimental month were conducted (Table A5). A significant difference in the mean weight of *B. albicinctus* adults and nymphs was detected between Trial 1 (commenced October) and Trial 2 (commenced December) (Welch two-sample test: *t* = −3.803, *df* = 126.79, *p* < 0.001). This difference was still detectable, albeit much weaker, when only adult *B. albicinctus* were considered (Welch two-sample test: *t* = −2.297, *df* = 67.854, *p* = 0.025). This indicated that adult *B. albicinctus* from the October cohort were on average 77.62% heavier than the December conspecifics. Statistically significant differences in insect weight were detected among *B. albicinctus* nymphs, adult males, and adult females (Kruskal–Wallis: χ^2^ = 25.141, *n* = 127, *p* < 0.001). The post hoc analysis revealed that *B. albicinctus* adult females were on average 53.67% heavier than adult males (Dunn test: *p* = 0.004). Nymphs, however, were heavier than both adult sexes: 65.64% heavier than adult males (Dunn test: *p* < 0.001) and 7.79% heavier than adult females (Dunn test: *p* < 0.001).

### 3.3. Relationships Between Measures of Insect Activity

**Figure 6:**
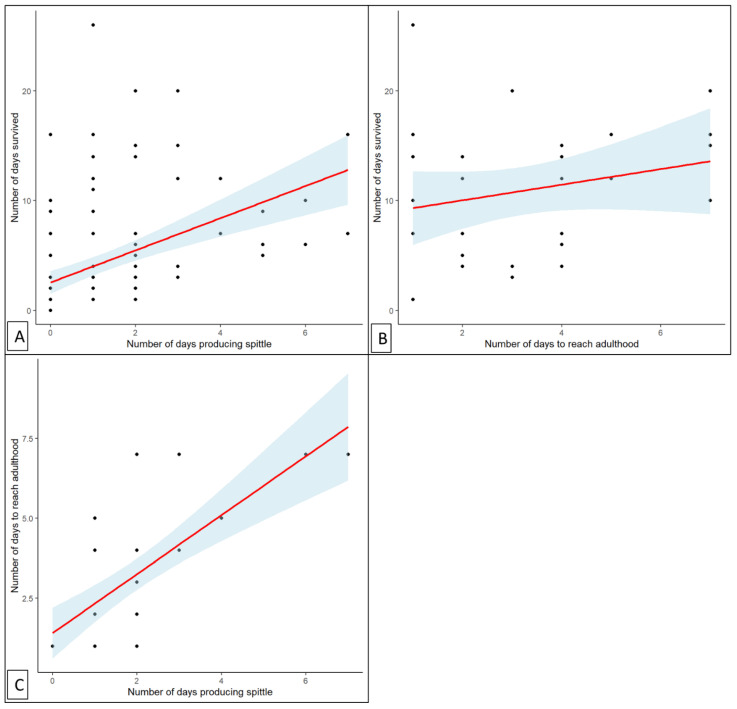
Scatterplots visualising the regression-modelled relationships (red line) between: (**A**) the number of days that *B. albicinctus* produced spittle and days survived, (**B**) the number of days until adulthood and the number of days survived, and (**C**) the number of days that *B. albicinctus* produced spittle and the number of days it took to reach adulthood. Light-blue shading either side of plotted model indicates 95% confidence interval of the mean.

The regression analysis revealed some associations between survival, spittle, and maturation measures (Table A6). A weak, but highly significant, positive association between the number of days survived and the number of days with spittle excretions was detected (linear regression: F_1,29_ = 30.44, *p* < 0.001, *r*^2^ = 0.197; Figure 6A). This indicated that, on average, for each day a *B. albicinctus* individual survived, the number of days spent excreting spittle increased by 0.13. This indicated that, for each additional day an insect produced spittle excretions, it would take an additional average of 0.59 days to reach adulthood. An association between insect longevity and the number of days to reach adulthood was not statistically supported by linear regression analysis (linear regression: F_1,29_ = 1.626, *p* = 0.212, *r*^2^ = 0.053; Figure 6B). The regression analysis also revealed that the number of days it took to reach the adult stage was moderately, and positively, correlated with the number of days with spittle excretions (linear regression: F_1,29_ = 34.89, *p* < 0.001, *r*^2^ = 0.546; Figure 6C). 

## 4. Discussion

This study provides clarification that the development and feeding activity of a possible *Xylella fastidiosa* vector, *Bathyllus albicinctus*, could differ in association with host plant and season-based insect cohorts. Statistical comparison revealed differences in insect weight between nymphs and adults, with nymphs found to weigh more than either male or female adult insects. This study also demonstrated that the species of host plant and the season insects began feeding influenced the survival, feeding, growth, and maturation of *B. albicinctus*. This provides an indication that the activity of *B. albicinctus*, and by association its capacity to possibly vector *X. fastidiosa*, might involve a combination of plant suitability as a host for *B. albicinctus* and the time of year. Further investigations could greatly enhance future *X. fastidiosa* control efforts by complementing this study with controlled field experiments, ideally with a focus on widespread and/or economically significant plant species that are known to be susceptible to both *B. albicinctus* and *X. fastidiosa*. This would provide confirmation that our findings can be generalised beyond the contained growth conditions used in this present study.

### 4.1. Insect Maturation in Spring and Summer

In this experiment we have identified variation in the feeding activity, longevity, and maturation timing of *B. albicinctus* nymphs feeding on one of four plant species. Nymphs matured into adults in both the October and December cohorts, which was partly consistent with other field studies on other aphrophorid Cercopoidea. *Philaenus spumarius* adults in Greek olive groves were reported to primarily emerge in spring, between March and May [27], but were not caught during summer months. Contrastingly, sampling from olive groves in Italy [28] caught *P. spumarius* adults during summer. These fluctuations in *P. spumarius* abundance were thought to correlate with the likelihood of successful *X. fastidiosa* transmission [18]. Like *P. spumarius*, the *B. albicinctus* life span, and therefore its opportunity to potentially transmit *X. fastidiosa,* could vary with life stage, sex, host and season. Our findings, while generally consistent with Cercopoidea elsewhere, were not highly replicated and were conducted under glasshouse laboratory conditions. To appropriately generalise these findings to field conditions, our study should be repeated in commercial growing conditions to better understand the role of seasonality in *B. albicinctus* development on host plants in the field.

### 4.2. Insect Maturation on Host Plants

Here we identified variation in the survival of *B. albicinctus* adults across a range of host plant species. Although insects could potentially mature into adults on all the host plants tested, our observations indicate that some host plants may only support nymph maturation into adults at certain times of year as adult maturation in *Bathyllus* appears to be highly seasonal. Despite this restriction, these host plants may still be susceptible to *X. fastidiosa* exposure if adult *B. albicinctus* carrying the bacteria attempt to feed on these plants. Additionally, our study did not explore insect choice, which does not always align with optimal development conditions (e.g., ecological traps) [29]. By the sheer number of plants in an environment, or through as-yet unknown factors perceived as attractive by the insect vector, some plants may be unsuitable for nymphal development while still being a real or dead-end food source for adult *B. albicinctus*. The interactions of optimal and preferred host plants, along with plant susceptibility to *X. fastidiosa* infection [16], will influence the risk of *X. fastidiosa* damage across different plant groups. Variable insect reproductive success between host plant species may also indicate variable risk in successful *X. fastidiosa* transmission. Due to a low concentration of organic compounds compared to other plant tissues [11,30], Cercopoidea, and some Cicadellinae, likely require considerable time feeding on live xylem vessels to facilitate insect growth. A further challenge for xylem-siphoning insects is the dangerously strong hydraulic force exerted by water moving vertically through xylem vessels, which can potentially render xylem feeding energetically non-viable [31]. The strength of these forces, and thus the effective availability of nutrients, is largely determined by environmental conditions that dictate stomatal activity [32,33,34]. Xylem activity in response to these environmental conditions will differ with the water availability and management strategies employed by different plant species [35,36]. Ultimately, this will have consequences for the development of many insect herbivores feeding on a water-stressed host plant [37,38,39].

**Figure 7:**
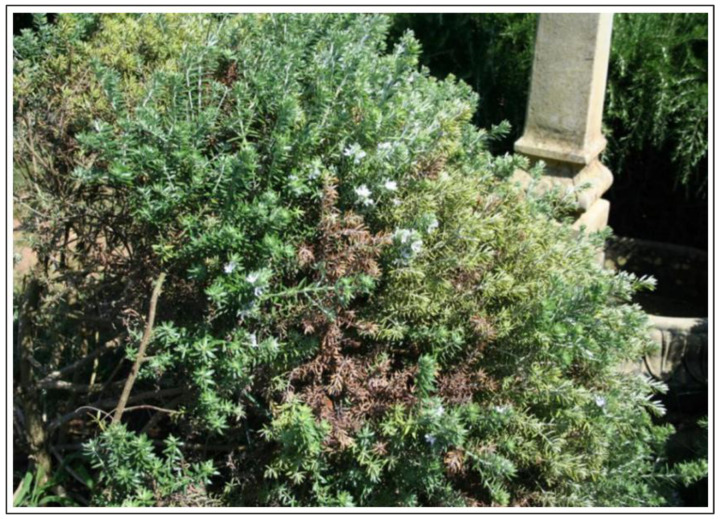
*Westringia fruticosa* infected with *Xylella fastidiosa* and exhibiting visible symptoms. Note the chlorotic and desiccated vegetation. Photo copyright held by Donato Boscia, CNR—Institute for Sustainable Plant Protection, UOS, Bari (Italy), European and Mediterranean Plant Protection Organisation.

As such, further investigations should focus on documenting the *X. fastidiosa* transmission potential of *B. albicinctus* on sub-optimal hosts, as well as known host plants of *B. albicinctus* that are also susceptible to *X. fastidiosa* (Figure 7). This could be explored with the use of an electropenetration graph that allows feeding activity in phloem- and xylem-feeding insects to be closely monitored in relation to pathogen transmission [40]. Our study of potential *X. fastidiosa* transmission dynamics in Australian Cercopoidea has highlighted the need to document the underlying feeding physiology of *B. albicinctus*, ideally on plants known to be susceptible to *X. fastidiosa*, such as olive and grapevine.

### 4.3. Host Suitability in Spring and Summer

The timing of insect collection was important for insect longevity, with the significantly heavier October-collected insects surviving up to 20 days and the lighter, and presumably younger, December-collected insects surviving up to 25 days. Feeding by *B. albicinctus* on grapevine, olive, oranges, and sow thistles suggests that these plants may be susceptible to *X. fastidiosa* infection by *B. albicinctus*, although their relative risk may differ. Despite taking great care when transplanting sow thistles, it is likely that these plants experienced elevated stress levels. Indeed, the herbaceous plants exhibited symptoms of low leaf water potential for several hours after transplantation, indicating difficulty in translocating essential water resources. This may partially explain the relatively poor success of *B. albicinctus* on sow thistle in our study [31]. *Bathyllus albicinctus* feeding on olives were the most likely to produce spittle and reach adulthood, with this group surviving the longest. Thus, *B. albicinctus* may have more opportunities to vector *X. fastidiosa* to olives than the other plant hosts in this study. This is consistent with observations in Italy, where *P. spumarius* and *P. italosignus* have been disproportionately responsible for infecting olive trees across the Apulia region [2,27,41]. Despite the detected variation between hosts, the success of *B. albicinctus* did not neatly align with the taxonomic relationship of host plants, and the observed differences between host plant groups were inconsistent between the spring and summer cohorts. This indicates that, at least for polyphagous *B. albicinctus*, the influence of host taxonomy for insect herbivore activity may be eclipsed by the season and/or climate conditions in which *B. albicinctus* and a host plant best develop [42,43]. Further investigations into the relationship between *B. albicinctus* and specific measures of environmental conditions (e.g., temperature) would allow for predictions of when and where this insect’s activity is greatest and, by association, the risk of potentially spreading diseases such as *X. fastidiosa*.

### 4.4. Correlations of Insect Activity Measures

In addition to season and host-based differences in insect activity, *B. albicinctus* that produced spittle excretions (indicative of feeding) generally survived for more days but also took longer to reach their adult stage. Insects that reached adulthood earlier did so with fewer feeding days, potentially because their feeding activity was more efficient. This feeding efficiency may be influenced by insect fitness or host quality [2]. Alternatively, perhaps *B. albicinctus* prolonged feeding and maturation to maximise their opportunity to accumulate more resources before moving to the reproductive adult stage [44]. Nevertheless, the deviation in adult emergence based on season (or calendar month) suggests that seasonal or climate cues are crucial in the development of *B. albicinctus*. The interpretation of these cues by *B. albicinctus* likely varies with the individual host plant responses to those same environmental cues [45]. Alternatively, sap-sucking insect development is greatly influenced by environmental conditions acting directly on ectothermic insects [46]. Yet, their development is potentially more impacted by the effect of environmental conditions on the host plant, thereby altering its activity and quality as a host for sap-sucking Hemiptera. Indeed, the climate experience of a host plant may be more important for the development of both phloem-feeding [47] and xylem-feeding insects [31] due to the importance of climate conditions (e.g., temperature, rainfall, and solar exposure) for phloem and xylem function [48,49]. Providing access to a natural unfiltered light cycle for host plants is incredibly important for encouraging plant activity and, by extension, a realistic level of attractiveness and suitability for *B. albicinctus* herbivores. Further investigation of *B. albicinctus* would therefore benefit from assessing insect growth under field conditions. This would allow for improved insight into potential *X. fastidiosa* transmission by better representing the growth dynamics of potential vectors, like *B. albicinctus*, feeding on field olive, grapevine, or orange crops.

## 5. Conclusions

With evidence of host and seasonal variation affecting *B. albicinctus* development, this study provides an indication of the factors that influence variation in *B. albicinctus* abundance. This has implications for the density and occurrence of *B. albicinctus* and, by association, the risk of vectoring xylem-propagating pathogens, such as *X. fastidiosa*, if it were to arrive in Australia. The rising threat of *X. fastidiosa* globally elevates the urgency to better understand and predict the activity of known and putative insect vectors, namely xylem-feeding Hemiptera. Environmental drivers of activity in xylem-feeding insects, and their host plants, are very important for successful transmission of *X. fastidiosa*. Therefore, documenting the seasonal abundance and driving factors of Australian cercopoid activity is a key step in preparing Australia’s commercial crop growers for a *X. fastidiosa* incursion. As *X. fastidiosa* is a serious threat to global plant health, we encourage researchers elsewhere to investigate the abundance and host preferences of local xylem-feeding insects to better understand the potential vectors of *Xylella* and inform management responses, ideally before the pathogen arrives in an area.

## Figures and Tables

**Table 1:** The number of plants and insects examined for both Trial 1 (October start) and Trial 2 (December start) to evaluate host use of *Bathyllus albicinctus*.

Host Plant	Variety	Number of Plants	Number of Insects per Plant	Total Number of Insects per Variety
Grapevine	Dawn Seedless	1	2	2
	Early Muscat	6	2	12
	Fiesta	1	2	2
	Thomuscat	1	2	2
Olive	Manzanillo	4	2	8
	Kalamata	6	2	12
Orange	Washington Navel	10	2	20
Sow Thistle	unidentified	1	4	4

## Data Availability

Data analysed in this study can be found in the Supplementary Materials.

## Supplementary Materials

The following supporting information can be downloaded at: https://www.mdpi.com/article/10.3390/biology15110886/s1.

## Appendix A

**Table A1:** Summary of Kruskal–Wallis tests of independent groups between host plant species. Statistically significant tests (*p* ≤ 0.05) are bolded.

Category	Insect Measure	Kruskal–Wallis Chi^2^ (*F*)	Sample Size (N)	Degrees of Freedom (*df*)	Level of Significance (*p*)
Host plant species	Days survived	6.3544	122	3	0.1
	**Days producing spittle**	**9.7479**	**122**	**3**	**0.02**
	Days to adult	1.8289	27	3	0.61

**Table A2:** Summary of pairwise Dunn test post hoc comparisons of the number of days insects produced spittle between host plants. Statistically significant pairs are bolded; Dunn test statistically significant when *p* ≤ 0.025 (α/2).

Host 1; Host 2	Mean Difference (Days) (Host 1–Host 2)	Level of Significance (*p*)	Host 1 Sample Size (n)	Host 2 Sample Size (n)
**Olive; Grapevine**	**2.752**	**0.003**	**40**	**38**
**Olive; Citrus-Orange**	**2.466**	**0.007**	**40**	**40**
Olive; Sow Thistle	1.701	0.044	40	8
Grapevine; Citrus-Orange	−0.317	0.376	38	40
Grapevine; Sow Thistle	0.091	0.464	38	8
Citrus-Orange; Sow Thistle	0.277	0.391	40	8

**Table A3:** Summary of Kruskal–Wallis tests of independent groups. Sample sizes were equal to 125 and degrees of freedom were equal to three. All tests of significant differences between host plant species within insect trial groups (i.e., Trials 1 (October) and 2 (December)) are summarised here. Statistically significant tests (*p* ≤ 0.05) are bolded.

Category	Insect Measure	Kruskal–Wallis Chi^2^ (*F*)	Sample Size (N)	Degrees of Freedom (*df*)	Level of Significance (*p*)
Trial 1 host plant species	Days survived	7.4401	58	3	0.06
	**Days producing spittle**	**7.836**	**58**	**3**	**0.05**
	Days to adult	0.603	9	2	0.74
Trial 2 host plant species	Days survived	3.7115	60	3	0.29
	Days producing spittle	7.1715	60	3	0.07
	Days to adult	1.5501	16	2	0.46

**Table A4:** Summary of pairwise Dunn test post hoc comparisons of the number of days insects produced spittle between host plants in Trial 1 (October). Statistically significant pairs are bolded; Dunn test statistically significant when *p* ≤ 0.025 (α/2).

October Host 1; October Host 2	Mean Difference (Days) (Host 1–Host 2)	Level of Significance (*p*)	October Host 1 Sample Size (n)	October Host 2 Sample Size (n)
**Olive; Grapevine**	**2.524**	**0.006**	**20**	**18**
Olive; Citrus-Orange	1.863	0.312	20	20
Olive; Sow Thistle	0.077	0.469	20	4
Grapevine; Citrus-Orange	−0.711	0.239	18	20
Grapevine; Sow Thistle	1.560	0.059	18	4
Citrus-Orange; Sow Thistle	−1.153	0.124	20	4

**Table A5:** Summary of Welch’s test between independent samples. Statistically significant differences (*p* ≤ 0.05) are bolded.

Category	Insect Measure	Critical Statistic (*t*)	Degrees of Freedom (*df*)	Level of Significance (*p*)	Trial 1 Sample Size (n)	Trial 2 Sample Size (n)
Starting month	**Adult weight**	**−2.297**	**67.854**	**0.0247**	**62**	**24**
	Days survived	1.122	123.41	0.2639	62	64
	Days to adult	−0.0534	22.471	0.9579	12	19
	Days producing spittle	−0.2461	122.91	0.806	62	64

**Table A6:** Summary of regression models used to determine if measures of insect survival and development were correlated with one another. Table includes model sample size (*N*), fit of the data (F), level of statistical significance (*p*), correlation strength (*r*^2^), residual mean square error (MSE), and square root of MSE (√ (MSE)). Statistically significant models (*p* ≤ 0.05) are bolded. Degrees of freedom were always one.

Measure 1	Measure 2	Model Equation	Critical Statistic (*F*)	Sample Size (N)	Level of Significance (*p*)	Correlation Strength (*r*^2^)	MSE	√ (MSE)
**Days survived**	**Days producing spittle**	**Y = 1.4647*x* + 2.5420**	**30.44**	**124**	**1.919 × 10^−7^**	**0.1971**	**21.6**	**4.648**
Days survived	Days to adult	Y = 0.7113*x* + 8.6086	1.626	29	0.2123	0.0531	33.01	5.745
**Days producing spittle**	**Days to adult**	**Y = 0.59272*x* + 0.05575**	**34.89**	**29**	**2.059 × 10^−6^**	**0.5461**	**1.068**	**1.0337**
