# Supplementary material for: Host Plant Preferences and Survival of the Native Australian Spittlebug, Bathyllus albicinctus Erichson (Hemiptera: Cercopoidea)
Source: Biology (Basel). 2026 Jun 3;15(11):886. doi: 10.3390/biology15110886 (PMC13255721; doi:10.3390/biology15110886)
Supplement: Supplementary file 1 [file biology-15-00886-s001.zip › biology-4318594-supplementary.pdf]

Table S1.

Collected Insect information		
StartMonth	Gender	Weight(g)
Dec	F	0.0037
Dec	F	0.0051
Dec	F	0.0046
Dec	F	0.0035
Dec	F	0.0051
Dec	F	0.0061
Dec	F	0.004
Dec	F	0.0063
Dec	F	0.0035
Dec	F	0.0046
Dec	F	0.0045
Dec	M	0.0023
Dec	M	0.0023
Dec	M	0.003
Dec	M	0.0026
Dec	M	0.0013
Dec	M	0.0048
Dec	M	0.0024
Dec	M	0.0031
Dec	M	0.002
Dec	M	0.0023
Dec	M	0.0022
Dec	M	0.0039
Dec	M	0.0025
Oct	N	0.001
Oct	N	0.0098
Oct	N	0.0083
Oct	N	0.0059
Oct	N	0.0071
Oct	N	0.001
Oct	N	0.0093
Oct	N	0.0081
Oct	N	0.0072
Oct	N	0.0067
Oct	N	0.0073
Oct	N	0.005
Oct	N	0.0033
Oct	N	0.0071
Oct	N	0.0125
Oct	N	0.0018
Oct	N	0.0101
Oct	N	0.0054

Oct	N	0.0077
Oct	N	0.0067
Oct	N	0.0099
Oct	N	0.0062
Oct	N	0.0084
Oct	N	0.0069
Oct	N	0.0035
Oct	N	0.0043
Oct	N	0.0072
Oct	N	0.0056
Oct	N	0.0099
Oct	N	0.0082
Oct	N	0.0084
Oct	N	0.0083
Oct	N	0.0099
Oct	N	0.0039
Oct	N	0.0062
Oct	N	0.0073
Oct	N	0.0033
Oct	N	0.0064
Oct	N	0.0059
Oct	F	0.0054
Oct	F	0.0051
Oct	F	0.0053
Oct	F	0.0053
Oct	F	0.0077
Oct	F	0.0052
Oct	F	0.0053
Oct	F	0.0052
Oct	M	0.0053
Oct	M	0.0061
Oct	M	0.0118
Oct	M	0.0037
Oct	M	0.0036
Oct	M	0.0047
Oct	M	0.0066
Oct	N	0.0041
Oct	N	0.0042
Oct	N	0.0036
Oct	N	0.0049
Oct	N	0.0081
Oct	F	0.0071
Oct	F	0.074
Oct	F	0.0034
Oct	F	0.0039
Oct	F	0.0064

Oct	F	0.0046
Oct	F	0.0042
Oct	F	0.0046
Oct	F	0.007
Oct	F	0.0028
Oct	F	0.0034
Oct	F	0.0042
Oct	F	0.0027
Oct	F	0.0045
Oct	F	0.0054
Oct	F	0.0051
Oct	F	0.0055
Oct	F	0.0051
Oct	F	0.0055
Oct	F	0.0051
Oct	F	0.0038
Oct	F	0.0042
Oct	F	0.0037
Oct	F	0.0047
Oct	F	0.0063
Oct	F	0.0065
Oct	F	0.0046
Oct	F	0.0035
Oct	F	0.0045
Oct	F	0.0054
Oct	F	0.005
Oct	F	0.0067
Oct	F	0.0048
Oct	F	0.0072
Oct	F	0.0041
Oct	F	0.0046
Oct	F	0.004
Oct	F	0.0048
Oct	F	0.0038
Oct	F	0.0042
Oct	F	0.0052
Oct	F	0.0054
Oct	F	0.0053
Oct	F	0.0074
Oct	F	0.0061
Oct	M	0.0056
Oct	M	0.0042

Table S2.

Insect survival on plants								
Plant ID	Start Month	Host	Host x Month	Cultivar	Insect ID	Survived Days	Spittle Days	Adult Days
1	October	Olive	OctOliv	Manzanillo	1	5	2	2
1	October	Olive	OctOliv	Manzanillo	2	16	7	7
2	October	Olive	OctOliv	Manzanillo	3	0	0	
2	October	Olive	OctOliv	Manzanillo	4	1	1	
3	October	Olive	OctOliv	Manzanillo	5	1	2	1
3	October	Olive	OctOliv	Manzanillo	6	12	3	4
4	October	Olive	OctOliv	Manzanillo	7	1	0	
4	October	Olive	OctOliv	Manzanillo	8	2	1	
5	October	Olive	OctOliv	Kalamata	9	4	2	2
5	October	Olive	OctOliv	Kalamata	10	0	0	
6	October	Olive	OctOliv	Kalamata	11	3	3	
6	October	Olive	OctOliv	Kalamata	12	15	3	4
7	October	Olive	OctOliv	Kalamata	13	1	0	
7	October	Olive	OctOliv	Kalamata	14	1	0	
8	October	Olive	OctOliv	Kalamata	15	14	1	2
8	October	Olive	OctOliv	Kalamata	16	1	1	
9	October	Olive	OctOliv	Kalamata	17	14	2	1
9	October	Olive	OctOliv	Kalamata	18	12	1	2
10	October	Olive	OctOliv	Kalamata	19	4	3	
10	October	Olive	OctOliv	Kalamata	20	15	2	7
11	October	Grapevine	OctGrap	Early Muscat	21	1	0	
11	October	Grapevine	OctGrap	Early Muscat	22	1	1	
12	October	Grapevine	OctGrap	Early Muscat	23	2	2	
12	October	Grapevine	OctGrap	Early Muscat	24	1	1	
13	October	Grapevine	OctGrap	Early Muscat	25	0	0	
13	October	Grapevine	OctGrap	Early Muscat	26	1	0	
14	October	Grapevine	OctGrap	Early Muscat	27	0	0	
14	October	Grapevine	OctGrap	Early Muscat	28	1	0	
15	October	Grapevine	OctGrap	Early Muscat	29	0	0	
15	October	Grapevine	OctGrap	Early Muscat	30	7	7	
17	October	Grapevine	OctGrap	Early Muscat	31	0	0	
17	October	Grapevine	OctGrap	Early Muscat	32	20	2	3

18	October	Grapevine	OctGrap	Dawn Seedless	33	1	1	
18	October	Grapevine	OctGrap	Dawn Seedless	34	1	0	
19	October	Grapevine	OctGrap	Thomuscat	35	2	0	
19	October	Grapevine	OctGrap	Thomuscat	36	0	0	
20	October	Grapevine	OctGrap	Fiesta	37	0	0	
20	October	Grapevine	OctGrap	Fiesta	38	2	1	
21	October	Citrus-orange	OctOran	Washington Navel	39	1	1	
21	October	Citrus-orange	OctOran	Washington Navel	40	0	0	
22	October	Citrus-orange	OctOran	Washington Navel	41	9	5	
22	October	Citrus-orange	OctOran	Washington Navel	42	2	1	
23	October	Citrus-orange	OctOran	Washington Navel	43	0	0	
23	October	Citrus-orange	OctOran	Washington Navel	44	2	0	
24	October	Citrus-orange	OctOran	Washington Navel	45	2	1	
24	October	Citrus-orange	OctOran	Washington Navel	46	3	0	
25	October	Citrus-orange	OctOran	Washington Navel	47	9	1	
25	October	Citrus-orange	OctOran	Washington Navel	48	11	1	
26	October	Citrus-orange	OctOran	Washington Navel	49	2	1	
26	October	Citrus-orange	OctOran	Washington Navel	50	1	1	
27	October	Citrus-orange	OctOran	Washington Navel	51	1	0	
27	October	Citrus-orange	OctOran	Washington Navel	52	2	1	
28	October	Citrus-orange	OctOran	Washington Navel	53	1	0	
28	October	Citrus-orange	OctOran	Washington Navel	54	3	0	
29	October	Citrus-orange	OctOran	Washington Navel	55	0	0	
29	October	Citrus-orange	OctOran	Washington Navel	56	4	1	
30	October	Citrus-orange	OctOran	Washington Navel	57	3	3	

30	October	Citrus-orange	OctOran	Washington Navel	58	2	1	
31	October	Thistle	OctThis	Thistle	59	6	2	4
31	October	Thistle	OctThis	Thistle	60	1	1	
31	October	Thistle	OctThis	Thistle	61	6	6	
31	October	Thistle	OctThis	Thistle	62	0	0	
1	December	Olive	DecOliv	Manzanillo	1	4	2	3
1	December	Olive	DecOliv	Manzanillo	2	6	5	
2	December	Olive	DecOliv	Manzanillo	3	3	2	3
2	December	Olive	DecOliv	Manzanillo	4	2	1	
3	December	Olive	DecOliv	Manzanillo	5	10	6	7
3	December	Olive	DecOliv	Manzanillo	6	3	1	
4	December	Olive	DecOliv	Manzanillo	7	3	3	
4	December	Olive	DecOliv	Manzanillo	8	3	3	
5	December	Olive	DecOliv	Kalamata	9	14	1	2
5	December	Olive	DecOliv	Kalamata	10	10	0	1
6	December	Olive	DecOliv	Kalamata	11	3	3	
6	December	Olive	DecOliv	Kalamata	12	12	3	4
7	December	Olive	DecOliv	Kalamata	13	1	0	
7	December	Olive	DecOliv	Kalamata	14	0	0	
8	December	Olive	DecOliv	Kalamata	15	7	0	1
8	December	Olive	DecOliv	Kalamata	16	0	0	
9	December	Olive	DecOliv	Kalamata	17	7	2	4
9	December	Olive	DecOliv	Kalamata	18	0	0	
10	December	Olive	DecOliv	Kalamata	19	7	1	2
10	December	Olive	DecOliv	Kalamata	20	2	1	
11	December	Grapevine	DecGrap	Early Muscat	21	2	0	
11	December	Grapevine	DecGrap	Early Muscat	22	2	0	

12	December	Grapevine	DecGrap	Early Muscat	23	2	0	
12	December	Grapevine	DecGrap	Early Muscat	24	5	0	
13	December	Grapevine	DecGrap	Early Muscat	25	1	0	
13	December	Grapevine	DecGrap	Early Muscat	26	1	1	
14	December	Grapevine	DecGrap	Early Muscat	27	9	0	
14	December	Grapevine	DecGrap	Early Muscat	28	0	0	
15	December	Grapevine	DecGrap	Early Muscat	29	1	1	
15	December	Grapevine	DecGrap	Early Muscat	30	20	3	7
16	December	Grapevine	DecGrap	Early Muscat	31	16	1	5
16	December	Grapevine	DecGrap	Early Muscat	32	1	0	1
17	December	Grapevine	DecGrap	Early Muscat	33	26	1	1
17	December	Grapevine	DecGrap	Early Muscat	34	0	0	
18	December	Grapevine	DecGrap	Dawn seedless	35	1	1	
18	December	Grapevine	DecGrap	Dawn seedless	36	4	2	4
19	December	Grapevine	DecGrap	Thomuscat	37	12	4	5
19	December	Grapevine	DecGrap	Thomuscat	38	2	2	
20	December	Grapevine	DecGrap	Fiesta	39	7	1	4
20	December	Grapevine	DecGrap	Fiesta	40	4	1	
21	December	Citrus-orange	DecOran	Washington navel	41	2	0	
21	December	Citrus-orange	DecOran	Washington navel	42	9	5	
22	December	Citrus-orange	DecOran	Washington navel	43	14	2	4
22	December	Citrus-orange	DecOran	Washington navel	44	0	0	
23	December	Citrus-orange	DecOran	Washington navel	45	2	0	
23	December	Citrus-orange	DecOran	Washington navel	46	2	0	
24	December	Citrus-orange	DecOran	Washington navel	47	2	2	

24	December	Citrus-orange	DecOran	Washington navel	48	2	0	
25	December	Citrus-orange	DecOran	Washington navel	49	5	2	
25	December	Citrus-orange	DecOran	Washington navel	50	0	0	
26	December	Citrus-orange	DecOran	Washington navel	51	14	1	2
26	December	Citrus-orange	DecOran	Washington navel	52	7	4	
27	December	Citrus-orange	DecOran	Washington navel	53	1	1	
27	December	Citrus-orange	DecOran	Washington navel	54	2	0	
28	December	Citrus-orange	DecOran	Washington navel	55	2	0	
28	December	Citrus-orange	DecOran	Washington navel	56	1	0	
29	December	Citrus-orange	DecOran	Washington navel	57	2	0	
29	December	Citrus-orange	DecOran	Washington navel	58	1	0	
30	December	Citrus-orange	DecOran	Washington navel	59	16	0	1
30	December	Citrus-orange	DecOran	Washington navel	60	5	5	
31	December	Thistle	DecThis	Thistle	61	1	0	
31	December	Thistle	DecThis	Thistle	62	1	0	
31	December	Thistle	DecThis	Thistle	63	2	0	
31	December	Thistle	DecThis	Thistle	64	1	0	
